# Genetic variant of the renin-angiotensin system and prevalence of type 2 diabetes mellitus: a modest but significant effect of aldosterone synthase

**DOI:** 10.1007/s00592-014-0561-7

**Published:** 2014-02-19

**Authors:** Mai Ichikawa, Tadashi Konoshita, Takahiro Nakaya, Katsushi Yamamoto, Mika Yamada, Satsuki Sato, Michiko Imagawa, Yasukazu Makino, Miki Fujii, Yasuo Zenimaru, Kenichiro Arakawa, Jinya Suzuki, Tamotsu Ishizuka, Hiroyuki Nakamura

**Affiliations:** 1 Third Department of Internal Medicine, Faculty of Medical Sciences, University of Fukui, 23-3, Matsuokashimoaizuki, Eiheiji, Fukui 910-1193 Japan; 2 Department of Environmental and Preventive Medicine, Kanazawa University Graduate School of Medical Science, Kanazawa, Japan

**Keywords:** Renin-angiotensin system, Gene polymorphism, Diabetes, Aldosterone, CYP11B2

## Abstract

Recent genome-wide association studies have identified multiple variants that confer risk of type 2 diabetes mellitus (DM). However, established associations explain only a part of the heritability. Thus, even at the genome-wide association studies era, candidate gene approach should be still useful. Recent interventional studies against the renin-angiotensin system (RAS) showed reduction in new onset of DM, implying the system is involved in the onset. We substantiated the hypothesis that genetic variants of RAS have significant association with prevalence of DM. We enrolled to the study consecutive 782 subjects who had consulted our hospitals for mainly lifestyle related diseases. They consisted of 282 (36.1 %) diabetes cases. Genotypes were assayed with genomic DNA for conventional four genes of the RAS, i.e., angiotensin converting enzyme (ACE) insertion/deletion variant, angiotensinogen (AGT) M235T variant, angiotensin II type I receptor (AT1) A1166C variant, and aldosterone synthase (CYP11B2) C-344T variant. Association between the genetic variants of the RAS and prevalence of type 2 DM was tested. A significant association of DM and CYP11B2 genotype was obtained. There was no significant association between DM and ACE, AGT and AT1 variants. A multivariate logistic regression showed that age, gender, and CYP11B2 genotype were independent factors for association to diabetes, the DM risk of CC/CT to TT being 1.40 (95 % CI 1.04–1.90, *p* = 0.029). Thus, it is concluded that a genetic variant of the RAS should have a modest but significant impact on the onset of type 2 diabetes mellitus.

## Introduction

In contrast to the standard candidate gene approach method, genome-wide association studies (GWAS), as one ultimate style of reverse genetics method, have been expected to identify considerable part of risk alleles and applied to diabetes (DM). Thus, recently, the GWAS have identified multiple loci containing variants that confer risk of type 2 diabetes mellitus [1–3]. However, established associations to common and rare variants explain only a part of the heritability of type 2 DM. This limit of GWAS is thought to be attributed to the so-called genome-wide significance level (*p* < 5 × 10^−8^). Thus, even at the GWAS era, candidate gene approach should be still useful to find new important responsible genes of poly genic disorders. The renin-angiotensin system (RAS) plays major roles in blood pressure regulation and electrolyte metabolism [4]; on the other hand, the over-activation of the RAS is thought to play pivotal roles in the pathophysiology of cardiovascular [5], renal [6], and metabolic conditions [7]. A sufficient number of clinical trials provided a firm effectiveness of blockade on the RAS against cardiovascular and renal conditions [8–10]. At the same time, the blockade showed the reduction in new onset of type 2 DM [11–13]. The genetic variants are also associated divers clinical aspects [14–16]. These observations are strongly implying that the RAS might be involved in the onset of DM itself.

Thus, in this study, we substantiated the hypothesis that genetic variants of the RAS have significant association with prevalence of type 2 DM.

## Methods

### Subjects and genotyping

We enrolled to the study consecutive 782 subjects who had consulted our hospitals for mainly lifestyle related diseases (hypertension, diabetes, dyslipidemia, and chronic kidney disease) with no special selection between June 2000 and July 2013. The study was undertaken in accordance with the 1975 Declaration of Helsinki Principles revised in 2008. The study was approved by the ethics committee of Fukui University (No. 13-1, 14-2), and written informed consent for participation was obtained from all individuals. Diabetes was diagnosed according to the criteria of the World Health Organization. Subjects with age <20 years old, type 1 diabetes, gestational diabetes, secondary diabetes, severe organ failure, and acute phase disorders were excluded. At the blood sampling, all subjects had been under the condition without any anti-hypertensive or anti-dyslipidemic agent at least 1 week. Diabetic subjects continued to receive their usual care for diabetes. The subjects consisted of 282 (36.1 %) diabetes cases and 365 (46.7 %) male cases. Mean age, body mass index (BMI), blood glucose level, glycosylated hemoglobin A1c, low-density lipoprotein, and estimated glomerular filtration rate (eGFR) were 63.0 ± 13.9 years, 23.9 ± 3.9, 119.7 ± 42.7 mg/dl, 6.36 ± 1.36 %, 116.7 ± 31.9 mg/dl, and 76.9 ± 22.5 ml/min/1.73 m^2^, respectively (Table 1).

**Table 1:** Baseline characteristics of subjects

Characteristics	
Number	782
Diabetes mellitus [no. (%)]	282 (36.1 %)
Male gender [no. (%)]	365 (46.7 %)
Age (year)	63.0 ± 13.9
Body mass index^a^	23.9 ± 3.9
Blood glucose (mg/dl)	119.7 ± 42.7
Glycosylated hemoglobin (%)	6.36 ± 1.36
Triglyceride (mg/dl)^b^	100.0 (68.0–143.0)
Cholesterol (mg/dl)	
High-density lipoprotein^b^	53.0 (44.0–63.0)
Low-density lipoprotein	116.7 ± 31.9
eGFR (ml/min/1.73 m^2^)	76.9 ± 22.5

Plus-minus values are mean ± SD

*eGFR* estimated glomerular filtration rate

^a^The body mass index is the weight in kilograms divided by square of the height in meters

^b^Values shown are medians (interquartile ranges)

Genotyping was carried out with genomic DNA isolated from human leukocytes by a commercial kit (QIAamp DNA Blood Mini Kit QIAGEN Inc., Japan). Angiotensinogen (AGT) M235T variant (rs699), [17] angiotensin II type I receptor (AT1) A1166C variant (rs5186) [18], and aldosterone synthase (CYP11B2) C-344T variant (rs1799998) [19] were assayed using the TaqMan method (Applied Biosystems, Foster city CA). Angiotensin converting enzyme (ACE) insertion/deletion (I/D) variant [20] was assayed by TaqMan method with special modification [21].

### Statistical analysis

The sample size of the study was calculated by setting the difference to be detected between gene groups at least 10 % of DM prevalence. By using χ^2^ analysis with 5 % of significance level and 80 % of power, it was calculated the study required 728 subjects in total. All statistical analyses were conducted using the SPSS version 11.0J (SPSS Japan, Inc., Japan). The allele frequencies for each genotype were tested by contingency table analysis. *p* < 0.05 was regarded as statistically significant. Data were presented as numbers, percentage, mean ± SD, or medians (interquartile ranges), as appropriate. The differences between two groups were analyzed by Student’s *t* test or Wilcoxon signed rank test as appropriate. Dichotomous characteristics were compared with use of the χ^2^ analysis for tests including Hardy–Weinberg equilibrium. Odds ratios for diabetes and 95 % confidential intervals were calculated using multivariate logistic regression for each dichotomous characteristic to adjust potential confounding factors.

## Results

Between non-diabetics and diabetics, we compared baseline characteristics of subjects (Table 2). Many items including age, gender, BMI, blood glucose, glycosylated hemoglobin, and lipids profiles showed significant difference between two groups.

**Table 2:** Comparison of baseline characteristics of subjects between non-diabetics and diabetics

Characteristics	DM (−)	DM (+)	*p* value^a^
Number	500	282	
Gender [(male) %]	41.8	55.7	<0.001
Age (≥65 year) (%)	45.2	55.0	0.005
Body mass index (≥23)^a^ (%)	53.8	60.4	0.047
Blood glucose (mg/dl)	101.0 ± 13.1	152.4 ± 55.1	<0.001
Glycosylated hemoglobin (%)	5.52 ± 0.41	7.51 ± 1.37	<0.001
Triglyceride (mg/dl)^b^	94.0 (65.0–135.5)	106.5 (76.75–159.0)	0.001
Cholesterol (mg/dl)			
High-density lipoprotein^b^	57.0 (47.0–65.0)	47.0 (41.0–58.0)	<0.001
Low-density lipoprotein	117.0 ± 31.7	116.2 ± 32.3	0.749
eGFR (ml/min/1.73 m^2^)	76.3 ± 19.5	77.8 ± 27.0	0.381

Plus-minus values are mean ± SD

*eGFR* estimated glomerular filtration rate

^a^The body mass index is the weight in kilograms divided by square of the height in meters

^b^Values shown are medians (interquartile ranges)

The numbers of individuals with each genotype were as follows: DD, ID, and II genotypes of ACE D/I were 111, 337, and 334, respectively; MM, MT, and TT genotypes of AGT M235T were 27, 236, and 519, respectively; AA, AC, and CC genotypes of AT1 A1166C were 672, 104, and 6, respectively; CC, TC, and TT genotypes of CYP11B2 C-344T were 70, 344, and 368, respectively. The distributions were similar to those expected from Hardy–Weinberg equilibrium. The distribution of diabetes status and genotypes of each RAS gene is shown in Table 3. Odds ratios for DM of each genotypes are as follows: About ACE (D/I) genotype, the risk of II to DD/DI was 1.186 [95 % confidence interval (CI) 0.884–1.592, *p* = 0.144]. About AGT (M235T) genotype, the risk of TT to MM/MT was 1.157 (95 % CI 0.848–1.5680, *p* = 0.200). About AT1 (A1166C) genotype, the risk of AA to AC/CC was 1.245 (95 % CI 0.810–1.916, *p* = 0.186). About CYP11B2 (C-344T) genotype, the risk of CC/CT to TT was 1.359 (95 % CI 1.012–1.824, *p* = 0.024). Thus, a significant association with DM was observed at aldosterone synthase variant with about 1.4 times risk.

**Table 3:** Distribution of genotypes of each gene and diabetes status

	N	ACE (D/I)		AGT (M235T)		AT1 (A1166C)		CYP11B2 (C-344T)	
		DD/DI	II	MM/MT	TT	AA	AC/CC	CC/CT	TT
DM (−)	500	294	206	174	326	425	75	251	249
DM (+)	282	154	128	89	193	247	35	163	119
OR (95 % CI) (risk for DM)		1.186 (0.884–1.592) (II to DD/DI)		1.157 (0.848–1.5680) (TT to MM/MT)		1.245 (0.810–1.916) (AA to AC/CC)		1.359 (1.012–1.824) (CC/CTA to CC)	
χ2		1.294		0.848		1.000		4.182	
*p* value		0.144		0.200		0.186		0.024	

*N* number, *ACE* angiotensin converting enzyme, *AGT* angiotensinogen, *AT1* angiotensin II type I receptor, *CYP11B2* aldosterone synthase, *DM* diabetes mellitus, *95* *% CI* 95 % confidence interval

Several confounding factors especially age effected on the results, finally a multivariate logistic regression was performed with 4 variables (Table 4). Considering that the dyslipidemia is attributed to metabolic disorder from diabetes, dyslipidemia was excluded from the final model of the analysis. Only age, gender, and CYP11B2 genotype were shown to be independent factors for association to diabetes. Thus, it was confirmed that a 1.4-fold increase in the odds for diabetes in subjects with C allele of CYP11B2 [Odds ratio 1.40 (95 % CI 1.04–1.90, *p* = 0.029)].

**Table 4:** Multivariate logistic regression analysis for diabetes

Variables	β	SE	Wald’s χ^2^	Odds Ratio (95 % CI)	*p* value
Gender (male, 41.8 %)	0.625	0.154	16.40	1.87 (1.38–2.53)	<0.001
Age (≥65 year, 48.7 %)	0.455	0.155	8.64	1.58 (1.16–2.14)	0.003
*CYP11B2* (CC/CT, 52.9 %)	0.337	0.154	4.77	1.40 (1.04–1.90)	0.029
Body mass index (≥23, 56.2 %)	0.202	0.161	1.57	1.22 (0.89–1.68)	0.211

Multivariate logistic regression was performed to adjust to potential confounding factor for diabetes. All factors in this table were included in the final model

*95* *% CI* 95 % confidence interval, *CYP11B2* aldosterone synthase

^a^ The body mass index is the weight in kilograms divided by square of the height in meters

## Discussion

It is well known that diverse factors affect onset of type 2 DM including so-called environmental and genetic ones. First, male gender has been known to be risk for the condition from cohort studies [22]. Age and obesity are also well known to be important environmental factors for type 2 DM [23, 24]. Our study also clearly indicated these factors are surely risk of type 2 DM in monovariate evaluations. Thus, we adjusted the confounding effects of these factors by multivariate logistic regression analysis. And it was well confirmed that the CYP11B2 variant is still independent risk of type 2 DM.

On general, for exploration of risk alleles for polygenic conditions, fundamentally two methods have been developed, candidate gene approach method and reverse genetics method including GWAS, one ultimate form of reverse genetics. The candidate gene approach is conducted to associations between genetic variations within genes of interest by known biological functions. On the contrary, GWAS scan the entire genome for common genetic variation without any biological foresight. Thus, GWAS is thought to be a strong tool for extract considerable part of causal genes unexpected from known pathophysiology; however, the obtained results do not always explain most part for type 2 DM [1–3]. This limit of GWAS is thought to be attributed to the so-called genome-wide strict significance level to avoid problems of multiple comparisons (*p* < 5 × 10^−8^). Thus, candidate gene approach is thought to be still useful to find responsible genes. Recent reports for the blockade of the RAS showed reduction in new onset of type 2 DM as sub-analysis, implying that the system might be involved in the onset of DM [11–13]. Plausible explanation has been made mainly by reduction in oxidative stress via the blockade of the system. Thus, the study substantiated the hypothesis that genetic variants of the RAS have significant association with prevalence of DM. So far, the association between the RAS genetic variants and the prevalence of type 2 DM has been examined in a small number of studies. Yang reported that interactions among RAS-related genes combination including ACE D/I polymorphism were associated with type 2 diabetes in a Chinese population [25]. A meta-analysis by Zhou reported that ACE I/D polymorphism is not associated with type 2 DM in a Chinese population [26]. As to CYP11B2, no report was made about the association with DM prevalence until now to our knowledge.

The C-344T is a promoter region polymorphism and thought to be a putative binding site for the steroidogenic transcription factor SF-1 [19] and might be associated with hypertension and salt sensitivity with inconsistency [27–32]. About the association between C-344T genetic variant and plasma aldosterone concentration is also controversial. Tamaki reported that the TC/CC genotypes were significantly associated with higher plasma aldosterone concentration/plasma renin activity [29]. However, majority of studies about plasma aldosterone concentration reported that the -344T allele was associated with higher plasma aldosterone levels [28, 31, 32]. In this context, it is thought to be plausible that the -344T allele might rather indicate a high prevalence of DM as presumed hypo-potassemic tendency by high plasma aldosterone. However, the results showed that the -344C allele tends to high DM prevalence. One strained possible explanation might be as follows: against the high plasma aldosterone tendency, plasma renin and angiotensin II might be reduced as a consequence of negative feedback and these reductions might lead to some anti-diabetic state. Thus, the result of the study should be difficult to explain clearly.

Several limitations of this study should be noted. We have calculated the sample size in consideration of type I error of 5 and 80 % of power; however, the sample number is still relatively small. Especially, the power calculation was not enough for the AT1 because of a small number for minor alleles 110 of 782 subjects. We evaluated 4 independent genetic variants. Accordingly, a correction for multiple testing might be needed. On this point, *p* value threshold 0.05 for significance might be relatively large. And one another limitation was that only one genetic variant for each gene was assayed.

In conclusion, this study provides a support that a genetic variant of CYP11B2 gene, C-344T is associated to prevalence of type 2 DM, in other words, the variant might be an independent predictor for the type 2 diabetes. Thus, a new possibility for personalized medicine by genetic variants of the renin-angiotensin system has been shown in the area of metabolic conditions. Further studies are necessary including other SNPs in each gene.
